# Renal Function in Ghanaian HIV-Infected Patients on Highly Active Antiretroviral Therapy: A Case-Control Study

**DOI:** 10.1371/journal.pone.0099469

**Published:** 2014-06-12

**Authors:** Christian Obirikorang, Derick Nii Mensah Osakunor, Benedict Ntaadu, Opei Kwafo Adarkwa

**Affiliations:** 1 Department of Molecular Medicine, School of Medical Sciences, College of Health Sciences, Kwame Nkrumah University of Science and Technology (KNUST), Kumasi, Ghana; 2 Department of Medical Laboratory Technology, Faculty of Allied Health Sciences, College of Health Sciences, Kwame Nkrumah University of Science and Technology (KNUST), Kumasi, Ghana; 3 Department of Obstetrics and Gynaecology, Komfo Anokye Teaching Hospital (KATH), Kumasi, Ghana.; University of Sao Paulo Medical School, Brazil

## Abstract

**Background:**

HAART is anticipated to result in an increase in long-term survival, but may present with the development of associated complications. The aim of this study was to assess the renal function of HIV-infected patients on antiretroviral therapy.

**Methods:**

A case-control study (January to May 2013) conducted at the Suntreso Government Hospital, Kumasi, Ghana. A total of 163 HIV-infected patients (mean age 39.9±10.22) were studied, of which 111 were on HAART (HIV-HAART) and 52 were not (HIV-Controls). Serum urea, creatinine, potassium, sodium, chloride and CD4 counts were measured with the determination of eGFR (CKD-EPI and MDRD). Data was analysed using GraphPad Prism. The Chi-square, t-test, one-way ANOVA and Spearman's correlation were used. *P* values <0.05 were considered significant.

**Results:**

Mean CD4 count of HIV-Controls was higher than that of HIV-HAART but was not significant (p = 0.304). But for sodium levels which were higher in HIV-Controls (p = 0.0284), urea (p = 0.1209), creatinine (p = 0.7155), potassium (p = 0.454) and chloride (p = 0.6282) levels did not differ significantly between both groups. All serum biochemical parameters did not differ significantly, irrespective of duration on therapy and CD4 counts. Based on regimen, sodium, chloride, urea and creatinine did not differ significantly between TDF, EVF and NVP-based therapies. Prevalence of CKD (eGFR <60 ml/min/1.73 m^2^) in the total population was 9.9% and 3.7% with the MDRD and EPI-CKD equations respectively.

**Conclusions:**

Renal insufficiency remains prevalent in HIV patients. Changes in renal function occur in HIV infection and over the course of HAART but the difference at either stage is not significant. This suggests the role of HIV infection, HAART and the presence of traditional risk factors but not HAART in itself, in renal dysfunction. We however recommend a close monitoring of patients before and during HAART, to aid in evaluating drug combinations and implement dose modifications when necessary.

## Introduction

The Human Immunodeficiency Virus (HIV) has been of immense concern over the years. It belongs to a larger group, the Retroviruses, based on mode of replication, and to a smaller group, the Lentiviruses, based on duration for onset of symptoms [1]. To help combat the infection, Highly Active Antiretroviral Therapy (HAART), a term that refers to the use of combinations of three or more antiretroviral agents was introduced. This has dramatically altered the treatment and life expectancy of HIV-infected individuals [2], [3].

Shortly after 1981 an entity now known as HIV-associated nephropathy (HIVAN), became evident and it remains the most common form of kidney disease among HIV-infected individuals [4], [5]. Studies have shown that there is significant renal impairment among HIV-infected patients who are naive to HAART [6]. In a recent study, a prospective analysis of 754 HIV-infected patients reported an incidence of 5.9 cases of acute renal failure per 100 patient-years [7]. In Ghana, it has been established that there is renal dysfunction among both patients on HAART and those yet to start HAART, and suggested a need for dose adjustments, especially before onset of therapy [8].

At all stages of HIV infection, renal disorders may be evident, ranging from fluid and electrolyte imbalances to end-stage renal disease (ESRD). Loss of kidney function may be attributable to treatment-related factors, intermittent viraemia, and traditional risk factors for kidney disease [5].

Improved survival among patients with HIV infection is anticipated to result in an increase in the long-term development of HAART-associated metabolic complications, such as diabetes and dyslipidemia, which in turn, can contribute to vascular changes and decreased renal function [9].

Metabolic alterations associated with HAART may also lead to significant elevations in serum lipid levels, accelerating the development of diabetes and the metabolic syndrome. These long-term metabolic changes will possibly cause an increase in diabetic and hypertensive renal disease as well as vascular complications [9].

Renal damage caused by antiretroviral drugs can result in a variety of toxic drug effects presenting as acute renal failure, tubular necrosis, kidney stones, or chronic renal disease [9]. Therefore, a plethora of comorbidities are often times present at the initiation of HAART or may emerge with the ageing of HAART-treated patients, thereby limiting therapeutic options and creating a therapeutic dilemma for clinicians.

Renal dysfunction may therefore be a common finding in patients infected with HIV, and necessitates increased surveillance and adaptation of dosages of HIV drugs [9]. Knowledge on the renal function status of such individuals from time to time, will improve management strategies.

The present study therefore aimed at assessing the renal function of HIV-infected patients on antiretroviral therapy.

## Methods

### Study Design/Site

A prospective case-control study, conducted from January to May 2013 at the Sexually Transmitted Infections (STI) clinic of the Suntreso Government Hospital, Kumasi, Ghana. A total of one hundred and sixty-three (163) HIV-infected participants were involved in the study, of which 111 were on HAART (designated as HIV-HAART) and 52 were not (designated as HIV-Controls). HIV-Controls were recruited such that there was no significant difference in age when compared to HIV-HAART individuals. To be included in the study, participants had to be diagnosed and confirmed HIV-patients, with ages ranging from 18 to 65 years.

### Ethical Consent

The study was approved by the Committee on Human Research Publication and Ethics (CHRPE) of the School of Medical Sciences (SMS), Kwame Nkrumah University of Science and Technology (KNUST) and Komfo Anokye Teaching Hospital (KATH) - CHRPE/AP/083/13. Written, informed consent was obtained from all participants, after detailed explanation of the study and its associated protocols.

### Sample Collection and Preparation

About 5 ml venous blood sample was taken from each participant. Three millilitres (3 ml) of the blood sample was dispensed into serum separator tubes (SST), allowed to clot and centrifuged at 3000rpm for 5 minutes. The serum was dispensed into eppendorf tubes and aliquots stored at −80°C until biochemical assays (urea and creatinine - Mindray BS 380 auto analyzer; sodium, potassium and chloride - Roche 9180 electrolyte analyzer) were performed. The remaining 2 ml of blood was put into ethylenediaminetetraacetic acid (EDTA) tubes for CD4 count estimation (BD FACS Count System-Becton Dickenson and Company, California, USA).

### Renal Function

The Chronic Kidney Disease Epidemiology Collaboration (CKD-EPI) and the Modification of Diet in Renal Disease (MDRD) study equations were used to assess renal function in HIV patients. The calculated estimated Glomerular Filtration Rate (eGFR) was used to stratify the study population into the five stages of Chronic Kidney Disease (CKD), based on the staging system of the Kidney Disease Outcomes Quality Initiative (K/DOQI). Stage 1 (Normal or increased eGFR)  =  ≥90 ml/min/1.73 m^2^; Stage 2 (Mildly decreased eGFR)  =  60–89 ml/min/1.73 m^2^; Stage 3 (Moderately decreased eGFR)  =  30–59 ml/min/1.73 m^2^; Stage 4 (Severely decreased eGFR)  =  15–29 ml/min/1.73 m^2^ and Stage 5 (Kidney failure)  =  <15 ml/min/1.73 m^2^.

The MDRD equation for creatinine in μmol/L: 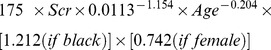



The CKD-EPI equation for creatinine in μmol/L:


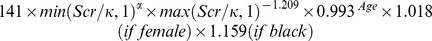


where *Scr* is serum creatinine in μmol/L, *k* is 61.9 for females and 79.6 for males, α is −0.329 for females and −0.411 for males, min indicates the minimum of *Scr*/*κ* or 1, and max indicates the maximum of *Scr*/*κ* or 1.

### Statistical Analysis

Data collected was sorted, coded, and entered into a Microsoft Excel spreadsheet for analysis using GraphPad Prism for Windows, version 5.0 (GraphPad Software, San Diego, CA, USA). Continuous variables were summarized by means ± (SD) and categorical variables were summarized by frequencies and percentages. The Chi-square, one-way ANOVA and student's t-test were used to compare variables, where appropriate. Spearman's correlation was used to determine the relationship between serum renal function parameters and duration on HAART. All *p* values <0.05 were considered significant.

## Results

A total of one hundred and sixty-three (163) participants were studied, with mean age, 39.9 ± 10.22 years and no significant difference in age between HIV-HAART and HIV-Controls (p = 0.5137). There were more females 127 (77.9%) than males 36 (22.1%) but this did not differ significantly when compared between HIV-HAART and HIV-Controls (p = 1.000). Most of the study participants were unmarried (60.7%) and involved in trading activities (44.8%). There was a significant variation in the kinds of occupation the participants were involved in (p = 0.0003). About 80% of participants had attained some level of education with most of them having had at least primary education (43.0%) [Table 1].

**Table 1:** Socio-demographic characteristics of study participants.

Parameter	Total (n = 163)	HIV-HAART (n = 111)	HIV-Controls (n = 52)	*p* value
Age				
Mean ± SD	39.9±10.22	40.26±10.219	39.13±10.290	0.5137
20–29 *n (%)*	32 (19.6)	20 (18.0)	12 (23.0)	0.8538
30–39 *n (%)*	53 (32.5)	36 (32.4)	17 (32.7)	
40–49 *n (%)*	43 (26.4)	31 (27.9)	12 (23.1)	
50–60 *n (%)*	35 (21.5)	24 (21.6)	11 (21.2)	
Sex				
Male *n (%)*	36 (22.1)	25 (22.5)	11 (21.2)	1.000
Female *n (%)*	127 (77.9)	86 (77.5)	41 (78.8)	
Marital status				
Married *n (%)*	64 (39.3)	41 (37.0)	23 (44.2)	0.3939
Unmarried *n (%)*	99 (60.7)	70 (63.1)	29 (55.8)	
Occupation				
Administrative *n (%)*	4 (2.5)	2 (1.8)	2 (3.8)	0.0003
Agricultural *n (%)*	31 (19.0)	23 (20.7)	8 (15.4)	
Trading *n (%)*	73 (44.8)	47 (42.3)	26 (50.0)	
Vocational *n (%)*	18 (11.0)	6 (5.4)	12 (23.1)	
Unemployed n (%)	37 (22.7)	34 (30.6)	3 (5.8)	
Education				
Illiterate *n (%)*	35 (21.5)	28 (25.2)	7 (13.5)	0.1758
Primary *n (%)*	70 (43.0)	48 (43.2)	22 (42.3)	
High School *n (%)*	55 (33.7)	34(30.6	21 (40.4)	
Tertiary *n (%)*	3 (1.8)	1 (0.9)	2 (3.8)	

n = Number. All p values <0.05 were considered significant.

Mean CD4 count of HIV-Controls was higher than that of HIV-HAART individuals but was not significant (p = 0.304). Potassium (p = 0.454) and chloride (p = 0.6282) levels did not differ significantly between HIV-HAART and HIV-Controls. Sodium levels were significantly higher in HIV-Controls when compared to HIV-HAART individuals (p = 0.0284). Urea and creatinine levels were higher among HIV-HAART individuals but was not significant (p = 0.1209, p = 0.7155 respectively) [Table 2].

**Table 2:** Serum biochemical and immunological characteristics of study participants.

Parameter	Total (n = 163)	HIV-HAART (n = 111)	HIV-Controls (n = 52)	*p* value
CD4 count (cells/μL)	522.6±288.4	506.6±245.27	556.6±364.3	0.3040
Sodium (mmol/L)	136.9±2.579	136.6±2.747	137.6±2.062	0.0284
Potassium (mmol/L)	4.058±0.3768	4.043±0.376	4.090±0.382	0.4540
Chloride (mmol/L)	97.68±7.027	97.86±7.67	97.29±5.471	0.6282
Urea (mmol/L)	3.427±1.226	3.530±1.106	3.210±1.434	0.1209
Creatinine (μmol/L	76.61±22.96	77.07±22.245	75.65±24.590	0.7155

All data are presented as mean ± SD. n = Number. p values <0.05 were considered significant.

Table 3 shows the serum biochemical parameters of HIV-HAART individuals, relative to gender. Although higher in males, mean sodium, potassium and chloride levels did not differ significantly with gender (p = 0.714, p = 0.252, p = 0.334 respectively). Urea (p = 0.0266) and creatinine (p = 0.0006) levels were significantly higher in males than females.

**Table 3:** Serum biochemical parameters of HIV-HAART participants stratified by gender.

Gender	Sodium (mmol/L)	Potassium (mmol/L)	Chloride (mmol/L)	Urea (mmol/L)	Creatinine (μmol/L)
Male (n = 25)	136.8±1.444	4.121±0.3706	99.21±3.451	3.971±1.040	90.60±19.90
Female (n = 86)	136.6±3.016	4.021±0.3770	97.49±8.459	3.407±1.099	73.29±21.47
*p* value	0.7144	0.2519	0.3336	0.0266	0.0006

All data are presented as mean ± SD. n = Number. p values <0.05 were considered significant.

Table 4 shows serum biochemical parameters of HIV-Controls, relative to gender. Similarly, mean sodium, potassium and chloride levels were higher in males but was not significant (p = 0.198, p = 0.247, p = 0.301 respectively). Like in HIV-HAART individuals, urea (p = 0.0178) and creatinine (p<0.0001) levels amongst HIV-Controls were higher in males than females.

**Table 4:** Serum biochemical parameters of HIV-Controls stratified by gender.

Gender	Sodium (mmol/L)	Potassium (mmol/L)	Chloride (mmol/L)	Urea (mmol/L)	Creatinine (μmol/L)
Male (n = 11)	138.3±1.348	4.209±0.2508	98.82±1.471	4.109±2.071	105.3±37.17
Female (n = 41)	137.4±2.188	4.059±0.4037	96.88±6.067	2.968±1.126	67.71±10.94
*p* value	0.1982	0.2465	0.3009	0.0176	< 0.0001

All data are presented as mean ± SD. n = Number. p values <0.05 were considered significant.

In Table 5, the effect of HAART duration on serum biochemical parameters is shown. Although there were changes in serum biochemical parameters over the course of HAART, parameters did not differ significantly, irrespective of HAART duration.

**Table 5:** Serum biochemical parameters of HIV-HAART participants stratified by duration on HAART.

Parameter	Below 1yr (n = 9)	1–2.9 yrs(n = 42)	3–4.9 yrs (n = 44)	≥5 yrs (n = 3)	*p* value
Sodium (mmol/L)	137.70±2.85	136.50±18.73	136.60±20.30	136.50±3.39	0.7518
Potassium (mmol/L)	4.06±0.81	4.10±2.59	4.016±2.59	3.93±0.54	0.4839
Chloride (mmol/L)	98.86±10.02	97.98±24.70	97.50±75.88	98.13±4.38	0.9715
Urea (mmol/L)	3.767±1.099	3.37±1.2576	3.557±1.027	4.4±0.1732	0.3997
Creatinine (μmol/L)	78.57±16.79	72.61±14.15	80.36±30.55	85.43±13.50	0.4209

All data are presented as mean ± SD. n = Number. p values <0.05 were considered significant.

A Spearman's correlation showed a non-significant positive correlation between duration on HAART, with sodium, urea and creatinine levels. Conversely, these parameters increase with ageing of HAART. There was however a non-significant negative correlation between HAART duration, potassium, and chloride levels [Table 6].

**Table 6:** Spearman's correlation of duration on HAART with serum biochemical parameters.

	Sodium	Potassium	Chloride	Urea	Creatinine
Duration on HAART (r)	0.014	−0.111	−0.013	0.090	0.159
*p* value	0.888	0.267	0.900	0.369	0.113

p values <0.05 were considered significant. r = correlation co-efficient.

But for potassium leves, participants on Tenofovir (TDF)-based therapy showed a trend of lower levels of biochemical parameters compared to those on Efavirenz (EFV) and Nevirapine (NVP)-based antiretroviral regimens. Levels of sodium, chloride, urea and creatinine did not differ significantly among particpants on different regimens. Potassium levels were highest amongst individuals on EVF-based regimen and differed significantly between the three groups compared (p = 0.0171) [Table 7].

**Table 7:** Serum biochemical parameters of HIV-HAART participants based on regimen.

Parameter	TDF-based	EFV-based	NVP-based	*p* value
Sodium (mmol/L)	133.5±3.536	136.7±2.083	136.6±3.609	0.2727
Potassium (mmol/L)	4.000±0.282	4.120±0.3783	3.908±0.3437	0.0171
Chloride (mmol/L)	89.00±14.14	97.99±9.271	98.10±2.326	0.2578
Urea (mmol/L)	2.700±1.273	3.626±1.131	3.403±1.056	0.3424
Creatinine(μmol/L)	57.45±6.293	78.91±26.05	74.80±13.12	0.2984

All data are presented as mean ± SD. TDF =  Tenofovir, EFV =  Efavirenz, NVP =  Nevirapine. p values <0.05 were considered significant.

Based on CD4 count categories (<200, 200 to 499 and ≥500 cells/uL), serum electrolytes did not differ amongst HIV-HAART individuals when compared to HIV-Controls [Table 8].

**Table 8:** Serum electrolytes of study participants stratified by CD4 count.

	CD4 <200 cells/uL			CD4 200–499 cells/uL		CD4 ≥500 cells/uL			
Parameter	HIV-Controls (n = 6)	HIV-HAART (n = 9)	*p* value	HIV-Controls (n = 19)	HIV-HAART (n = 48)	*p* value	HIV-Controls (n = 27)	HIV-HAART (n = 54)	*p* value
Sodium (mmol/L)	138.50 ±0.85	136.80±0.78	0.0839	137.3±0.52	136.80±0.36	0.2025	137.50±0.38	136.70±0.63	0.136
Potassium (mmol/L)	3.93±0.17	4.21±0.16	0.1351	4.163±0.08	4.054±0.05	0.1256	4.07±0.07	4.01 ±0.05	0.2264
Chloride (mmol/L)	99.33±0.33	95.22±2.05	0.0656	97.42±1.06	98.85±0.40	0.0612	96.74±1.26	98.32±0.43	0.1269

All data are presented as mean ± SD. n = Number. p values <0.05 were considered significant.

Table 9 compares the prevalence of renal dysfunction using the MDRD and CKD-EPI criteria. With the MDRD equation, prevalence of CKD (eGFR <60 ml/min/1.73 m^2^) was 9.9%. Thirty-two (28.8%) HIV-HAART individuals, had normal eGFR ≥90 ml/min/1.73 m^2^ but 68 (61.80%) had eGFR between 60–89 ml/min/1.73 m^2^ (mild renal impairment). Ten HIV-HAART individuals, representing 9.10%, had eGFR between 30–59 ml/min/1.73 m^2^ (moderate renal impairment), 1 (0.90%) had eGFR between 15–29 ml/min/1.73 m^2^ (severe renal impairment). No participant had renal failure, based on this equation or classification mode.

**Table 9:** Comparison of renal function amongst study participants (MDRD and CKD-EPI).

Parameter	Total (n = 162)	HIV-Controls (n = 52)	HIV-HAART (n = 111)	*p* value
*MDRD*				
Stage 1 (eGFR>90)	53 (32.70%)	22 (42.30%)	32 (28.8%)	0.1264
Stage 2 (eGFR 60–89)	93 (57.40%)	25 (48.10%)	68 (61.80%)	0.1256
Stage 3 (eGFR 30–59)	15 (9.30%)	5 (9.60%)	10 (9.10%)	1.0000
Stage 4 (eGFR 15–29)	1 (0.60%)	0 (0.00%)	1 (0.90%)	1.0000
Stage 5 (eGFR <15)	0 (0.00%)	0 (0.00%)	0 (0.00%)	−
*CKD-EPI*				
Stage 1 (eGFR>90)	99 (61.10%)	33 (63.50%)	67 (60.00%)	0.8365
Stage 2 (eGFR 60–89)	57 (35.20%)	17 (32.69%)	38 (34.50%)	0.8605
Stage 3 (eGFR 30–59)	5 (3.10%)	2 (3.85%)	5 (4.50%)	1.0000
Stage 4 (eGFR 15–29)	1 (0.60%)	0 (0.00%)	1 (0.90%)	1.0000
Stage 5 (eGFR <15)	0 (0%)	0 (0%)	0 (0%)	−

MDRD, Modification of Diet in Kidney Disease, CKD-EPI, Chronic Kidney Disease Epidemiology Collaboration. eGFR: estimated Glomerular Filtration Rate. n = Number. p values <0.05 were considered significant.

Using the CKD-EPI equation however, prevalence of CKD (eGFR <60 ml/min/1.73 m^2^) was 3.7%. A greater number, 67 (60.0%) of HIV-HAART individuals had normal renal function (≥90 ml/min/1.73 m^2^) with 38 (34.50%) having mild renal impairment. Five (4.50%) HIV-HAART individuals had eGFR between 30–59 ml/min/1.73 m^2^ (moderate renal impairment). Likewise, the same number of HIV-HAART individuals had severe renal impairment and no participant had renal failure.

Renal function however did not differ significantly between HIV-HAART and HIV-Controls based on both equations.

## Discussion

The present study describes the levels of various serum renal biochemical parameters as well as the assessment of renal function in HIV-infected individuals, on and off antiretroviral therapy. HIV patients increasingly require careful monitoring and evaluation for altered renal function, to prevent comorbidities of treatment and non-treatment-related nephrotoxicity. While HAART has dramatically changed treatment approach and increased longevity of HIV-infected individuals, various antiretroviral renal side effects are associated with different classes of antiretroviral combinations. In order to control HIV and non-HIV-related renal toxicities, renal function must be evaluated frequently and clinicians need to closely evaluate drug combinations to implement dose modifications when necessary.

Overall, we reported a high proportion of female HIV-infected individuals, but this did not differ significantly when compared between HIV-HAART and HIV-Controls. It is estimated that of the total number of people living with HIV/AIDS, women make up the majority. This is especially evident in countries with high HIV prevalence (including Lesotho, Namibia, and Zimbabwe), where an estimated three women are infected with HIV for every two men [10], [11]. Thus prevalence of HIV infection is higher by female proportion, hence the finding in the current study.

Furthermore, women are more likely to contract HIV, especially through vaginal intercourse than males [12], [13]. Factors such as early onset of sexual activity, sexually transmitted diseases, wider surface area of the vagina and longer semen-vaginal contact have been attributed to this [10]. With these factors apparent, the low level of education, occupation and hence standard of living may be a reflection of an increase in commercial sex workers, dominated by women, hence an increased prevalence of HIV-infection amongst women [10].

In addition, the total hospital attendance in Ghana is dominated by women, hence there is a higher likelihood for detection and diagnosis of various diseases and infections amongst women than in men. A theory which could explain this finding is that women are more likely to seek medical help than men. As such more women have the tendency to report to facilities even when they appear to be in good shape for routine checks where subclinical diseases and infections can be detected and diagnosed. It is assumed that men are more likely to employ a public reluctance to seek help as a way of demonstrating masculinity [14], hence data picked up in hospitals are more likely to be dominated by women.

Majority of participants in the present study were unmarried. Marital status has been reported to be associated with HIV seropositivity for both men and women and reports are that male-to-female ratio of HIV infection is often seen in the unmarried [15]. This is an indication that other factors may influence transmission and prevalence of HIV infection, especially in the unmarried. It is more likely that unmarried individuals have the tendency to engage in sexual activity at a younger age and with multiple partners, thereby reducing safe practices and increasing risk of HIV infection. In a joint study in Kenya and Zambia, the risk of HIV infection was most apparent in the younger population, the divorced and widowed. Prevalence was lowest amongst those who married as virgins and the risk of infection decreased after adjusting for marriage [15]. These suggest that safe practices are reduced and HIV is more prevalent amongst the unmarried, hence the finding in the present study.

We observed that about 80% of the study participants were educated. This is in line with reports that education results in the more educated less likely to be infected, a negative linear relationship between years of education and HIV [16], [17]. It is noteworthy that although the proportion of illiteracy was low in the present study, the level of attainment and years of study was also low. Education progressively increases the individual's capacity to process and understand information related to risks of HIV infection. Hence the higher the educational status, the higher the perceived risks and tendency to analyse behavioural choices and unmask potential risks [17]. Contrary findings have however been reported, where a higher level of attainment in education was associated with a low risk perception of HIV infection [18]. Thus effectiveness of eductaion may play a huge role in risk perception.

Occupation is known to affect HIV transmission and prevalence, especially in health care workers and occupations involving human and animal contact [19], [20]. In our case however, majority were involved in trading. Evidence suggests that street traders are restricted by lack of education and access to resources, and therefore remain at near-end survival. They also have poor occupational health and safety standards and face greater exposure to work-related risks including the heightened risk of ill-health [21]. To the best of our knowledge, there is little research that links work in the informal sector with HIV infection. However, one such conducted in Uganda found that women living and working in urban trading areas are at a far higher risk of HIV infection [22].

Upon HIV infection, CD4 counts are expected to begin dropping with time and HAART is expected to help the situation. Contrary to other studies, CD4 counts were lower in HIV-HAART individuals when compared to HIV-Controls. This could be due to the fact that most HIV-Controls, although with lowering levels of CD4 counts, did not have values so low as to initiate HAART. Studies have shown that HAART is associated with an increase in CD4 count [23]. Smith and colleagues demonstrated that, after 6, 12, and 24 months of HAART, the median increases in CD4 cell counts were 114, 181, and 248 cells/uL^3^, respectively. In the current study therefore, HIV-HAART individuals may have had increased CD4 counts upon initiation of HAART, but the slightly higher CD4 levels of HIV-Controls may have masked this. As such the possibility of an increase in CD4 counts, as demonstrated in other studies cannot be ruled out. Furthermore, our study did not focus on adherence monitoring and with others [24] having demonstrated that adherence to HAART is highly essential to the achievement of the purpose of the therapy, this may be a possible cause explanation to the findings in the present study.

Urea and creatinine levels were significantly higher in males than females. This is in line with standards and reference ranges established for both sexes for these analytes. Urea and creatinine levels are expected to be slightly higher in males than in females due to greater muscle mass and variations in metabolism, hence the finding in the present study [25].

We observed that irrespective of CD4 counts and duration of HAART, biochemical parameters did not differ between HIV-HAART and HIV-Controls. This is in line with a study conducted in Nigeria [5]. Okuonghae and colleagues established that there are no significant differences in renal function parameters in participants on and off HAART, when compared to normal healthy controls. They attributed this to the fact that sampling at the time of the study was such that infection was still in its early stages and thus renal damage was minimal [5]. In the present study, most of the participants had normal or minimal damage to renal function, based on classification criteria. This may be attributed to the absence of traditional risk factors like diabetes and high blood pressure, which are known to be associated with renal impairment [26]. Evidence from a study conducted to assess renal function among diabetics and HIV-infected individuals showed that diabetic patients exhibit greater electrolyte disturbances than people living with HIV/AIDS (HAART and non-HAART) [27].

Furthermore, the largely normal renal function in HIV-HAART individuals could be attributed to the fact that HAART, in attempts to bring down viral loads and increase CD4 counts, leads to a decrease in the rate of damage caused by HIV infection [5].

The present study showed that participants on Tenofovir (TDF)-based therapy had lower levels of serum renal biochemical parameters compared to those on Efavirenz (EFV) and Nevirapine (NVP)-based antiretroviral regimens. Similarly, studies have reported subclinical alteration of renal function in patients on TDF [28] and that TDF-related severe nephrotoxicity is an uncommon and reversible event, if it occurs at all [9], [29]. The observation in the present study could also be due to other factors such as hydration status and muscle mass which may influence such analytes.

Potassium levels differed significantly between the three groups compared (TDF, EFV and NVP). The mean levels observed in all groups of antiretroviral regimens were however within normal range [30]. The observed difference is indictive of the fact that HAART may be associated with significant changes in potassium levels and close monitoring may be warranted upon initiation of therapy.

Using the CKD-EPI equation, stage 2 renal insufficiency was 34.5% and 32.7% in HIV-HAART and HIV-Controls respectively. Previous studies have however reported higher incidences of stage 2 renal insufficiencies as 51.8%, [31] 55.8%, [32] and 48.5% [33]. Prevalence of stage 3 renal insufficiency (MDRD-9.60% and CKD-EPI-9.10%) in the present study is in agreement with findings in previous studies from other parts of the world [34], [35].

In the present study, the calculated prevalence of CKD (GFR <60 ml/min/1.73 m^2^; stages 3, 4 & 5) in the total population was 9.90% and 3.70% based on the MDRD and EPI-CKD equations respectively. In a related study, renal insufficiency in an antiretroviral (ARV) naive Kenyan population, [36] was 11.5% and 4.8% (CrCl <60 ml/min/1.73 m^2^ and <50 ml/min/1.73 m^2^ respectively). Despite being higher than that in the present study, our findings suggest that CKD is a growing health concern in this population, irrespective of therapy and caution must be exercised in the management of such patients.

In a similar study conducted in Ghana [8] a significant proportion of the Ghanaian HIV-infected population (9.8% males, 11.1% females) were found to have renal insufficiency and a dose adjustment was recommended upon initiation of therapy. This recommendation may have been adhered to in recent times and this reflected in the current study, with a lower proportion of HIV-HAART individuals with renal insufficiency. It is therefore evident that, provided dose recommendations and adjustments are adhered to for the majority of antiretroviral substances, HAART-related effects on renal function with regards to nephrotoxicity will be reduced [9].

## Conclusions

The findings in the present study demonstrates that renal insufficiency is still prevalent in HIV patients. Changes in renal function occur in HIV infection and over the course of HAART but the difference at either stage is not significant. This suggests the role of HIV infection, HAART and the presence of other traditional risk factors but not HAART in itself, in renal dysfunction. As suggested by others [8], [9] we recommend a close monitoring of patients before and during HAART to aid in evaluating drug combinations and implement dose modifications when necessary.

## References

[1] Bailes E, Gaa F, Chen Y (2003) Hybrid. Origin of SIV in Chimpanzees. Science 300: 1713–1725. 12805540 10.1126/science.1080657

[2] Palella FJJ, Delaney KM, Moorman AC, O LM, Fuhrer J, et al (1998) Declining morbidity and mortality among patients with advanced human immunodeficiency virus infection. HIV Outpatient Study Investigators. N Eng J Med 338: 853–860. 10.1056/NEJM199803263381301 9516219

[3] Berns JS, Kasbekar N (2006) Highly Active Antiretroviral Therapy and the Kidney: An Update on Antiretroviral Medications for Nephrologists. Clin J Am Soc Nephrol 1: 117–129. 17699198 10.2215/CJN.00370705

[4] Rao TK, Filippone EJ, Nicastri AD, Landesman SH, Frank E (1984) Associated focal and segmental glomerulosclerosis in the acquired immunodeficiency syndrome. N Eng J Med 310: 669–673. 10.1056/NEJM198403153101101 6700641

[5] Okuonghae PO, Olisekodiaka MJ, Onuegbu J, Amara AG, Aberare LO, et al (2011) Evaluation of renal function in HIV patients on antiretroviral therapy. Adv Lab Med Int 1: 25–31.

[6] Onodugo OD, Chukwuka C, Onyedum C, Ejim E, Mbah A, et al. (2013) Baseline Renal Function among Antiretroviral Therapy-Naive, HIV-Infected Patients in South East Nigeria. Journal of the International Association of Providers of AIDS Care (JIPAC). 10.1177/2325957413488169 23771870

[7] Franceschini N, Napravnik S, Eron JJJ, Szczech LA, Finn WF (2005) Incidence and etiology of acute renal failure among ambulatory HIV- infected patients. Kidney international 67: 1526–1531. 15780107 10.1111/j.1523-1755.2005.00232.x

[8] Owiredu WKBA, Quaye L, Amidu N, Addai-Mensah O (2013) Renal insufficiency in Ghanaian HIV infected patients: need for dose adjustment. African health sciences 13: 101–111. 23658575 10.4314/ahs.v13i1.14 PMC3645095

[9] Röling J, Schmid H, Fischereder M, Draenert R, Goebel FD (2006) HIV-Associated Renal Diseases and Highly Active Antiretroviral Therapy–Induced Nephropathy. Clinical Infectious Diseases 42: 1488–1495. 16619164 10.1086/503566

[10] (2002) Women and the AIDS epidemic: an update. HRSA careaction: 5–8. 12004768

[11] UNAIDS (2007) AIDS Epidemic Update. Geneva: UNAIDS.

[12] Comparison of female to male and male to female transmission of HIV in 563 stable couples. European Study Group on Heterosexual Transmission of HIV. BMJ 304: 809–813. 10.1136/bmj.304.6830.809 PMC1881672 1392708

[13] Padian NS, Shiboski SC, Jewell NP (1991) Female-to-male transmission of human immunodeficiency virus. JAMA: the journal of the American Medical Association 266: 1664–1667. 1886189

[14] Hunt K, Adamson J, Hewitt C, Nazareth I (2011) Do women consult more than men? A review of gender and consultation for back pain and headache. Journal of health services research & policy 16: 108–117. 20819913 10.1258/jhsrp.2010.009131 PMC3104816

[15] Glynn JR, Carael M, Auvert B, Kahindo M, Chege J, et al (2001) Why do young women have a much higher prevalence of HIV than young men? A study in Kisumu, Kenya and Ndola, Zambia. AIDS 15 Suppl 4S51–60. 11686466 10.1097/00002030-200108004-00006

[16] Smith W, Salinas D, Baker DP (2012) Multiple Effects of Education on Disease: The Intriguing Case of HIV/AIDS in Sub-Saharan Africa. In: A. W. Wiseman and R. N. Glover, editors. The Impact of HIV/AIDS on Education Worldwide (International Perspectives on Education and Society). Emerald Group Publishing Limited. pp. 79–104.

[17] Peters E, Baker DP, Dieckmann NF, Leon J, Collins J (2010) Explaining the effect of education on health: a field study in Ghana. Psychological science 21: 1369–1376. 20739672 10.1177/0956797610381506

[18] Essien EJ, Ogungbade GO, Ward D, Ekong E, Ross MW, et al (2007) Influence of educational status and other variables on human immunodeficiency virus risk perception among military personnel: a large cohort finding. Military medicine 172: 1177–1181. 18062392 10.7205/milmed.172.11.1177 PMC2137161

[19] Gumodoka B, Favot I, Berege ZA, Dolmans WM (1997) Occupational exposure to the risk of HIV infection among health care workers in Mwanza Region, United Republic of Tanzania. Bulletin of the World Health Organization 75: 133–140. 9185365 PMC2486935

[20] Haagsma JA, Tariq L, Heederik DJ, Havelaar AH (2012) Infectious disease risks associated with occupational exposure: a systematic review of the literature. Occupational and environmental medicine 69: 140–146. 22006935 10.1136/oemed-2011-100068

[21] Chen M, Jhabvala R, Lund F (2001) Supporting Workers in the Informal Economy: A Policy Framework. ILO, Geneva: ILO taskforce on the Informal Economy.

[22] Kirunga CT, Ntozi JP (1997) Socio-economic determinants of HIV serostatus: a study of Rakai District, Uganda. Health transition review: the cultural, social, and behavioural determinants of health 7 Suppl: 175–188 10169643

[23] Smith CJ, Sabin CA, Youle MS, Kinloch-de Loes S, Lampe JWFC, et al (2004) Factors Influencing Increases in CD4 Cell Counts of HIV-Positive Persons Receiving Long-Term Highly Active Antiretroviral Therapy. The Journal of infectious diseases 190: 1860–1868. 15499544 10.1086/425075

[24] Obirikorang C, Selleh PK, Abledu JK, Fofie CO (2013) Renal insufficiency in Ghanaian HIV infected patients: need for dose adjustment. ISRN AIDS 2013: 1–7.

[25] Finney H, Newman DJ, Price CP (2000) Adult reference ranges for serum cystatin C, creatinine and predicted creatinine clearance. Ann Clin Biochem 37: 49–59. 10672373 10.1258/0004563001901524

[26] Estrella MM, Parekh RS, Abraham A, Astor BC, Szczech LA, et al. (2010) The impact of kidney function at highly active antiretroviral therapy initiation on mortality in HIV-infected women. J Acquir Immune Defic Syndr 155: 217– 220. 10.1097/QAI.0b013e3181e674f4 PMC3243740 20581688

[27] Ugwuja E, Eze N (2006) A Comparative Study of Serum Electrolytes, Total Protein, Calcium and Phosphate Among Diabetic and HIV/AIDS Patients in Abakaliki, Southeastern, Nigeria. The Internet Journal of Laboratory Medicine Volume 2.

[28] Gallant JE, Moore RD (2009) Renal function with use of a tenofovir-containing initial antiretroviral regimen. AIDS 23: 1971–1975. 19696652 10.1097/QAD.0b013e32832c96e9 PMC3790472

[29] Gerard L, Chazallon C, Taburet AM, Girard PM, Aboulker JP, et al (2007) Renal function in antiretroviral-experienced patients treated with tenofovir disoproxil fumarate associated with atazanavir/ritonavir. Antivir Ther 12: 31–39. 17503745

[30] Abbassi-Ghanavati M, Greer LG, Cunningham FG (2009) Pregnancy and laboratory studies: a reference table for clinicians. Obstetrics and gynecology 114: 1326–1331. 19935037 10.1097/AOG.0b013e3181c2bde8

[31] Agaba EI, Agaba PA, Sirisena ND, Anteyi EA, JA I (2003) Renal Diseases in the Acquired Immunodeficiency Syndrome in North Central Nigeria. Nig J of Med 12: 120–125. 14737980

[32] Susman E (2005) Glomerular filtration rates seen as more sensitive measure of kidney disease in HAART-treated HIV patients. 12th conference on retroviruses and opportunistic infections (CROI). Boston, MA.

[33] Andia I, Pepper L, Matheison P (2005) Prevalence of renal disease in patients attending the HIV/AIDS clinic, at Mbarara University Teaching Hospital. 3rd IAS conf HIV Pathog Treat.

[34] Gupta SK, Mamlin BW, Johnson CS, Dollins MD, Topf JM, et al (2004) Prevalence of proteinuria and the development of chronic kidney disease in HIV-infected patients. Clinical nephrology 61: 1–6. 14964451 10.5414/cnp61001

[35] Chi YC, Kim MW, Man PL, Yan LL, Heidi K, et al. (2007) Prevalence of chronic kidney disease in Chinese HIVinfected patients. Nephrol Dialysis Transplantation 10.

[36] Wools-Kaloustian K, Gupta SK, Muloma E, Owino-Ong'or W, Sidle J, et al (2007) Renal disease in an antiretroviral-naive HIV-infected outpatient population in Western Kenya. Nephrology, dialysis, transplantation: official publication of the European Dialysis and Transplant Association - European Renal Association 22: 2208–2212. 10.1093/ndt/gfm223 17652119

