# Correction: Renal Function in Ghanaian HIV-Infected Patients on Highly Active Antiretroviral Therapy: A Case-Control Study

**DOI:** 10.1371/journal.pone.0111870

**Published:** 2014-10-21

**Authors:** The PLOS ONE Staff

There is an error in reference 24. The correct reference is: Obirikorang C, Selleh PK, Abledu JK and Fofie CO (2013) Predictors of Adherence to Antiretroviral Therapy among HIV/AIDS Patients in the Upper West Region of Ghana. ISRN AIDS 2013: 873939.

In Table 9, there is an error in the parameters for Stage 1 of MDRD and CKD-EPI. The parameters should read: Normal (eGFR≥90). There is an error in the parameters for Stage 2 of MDRD and CKD-EPI. The parameters should read: Mildly Reduced (eGFR 60-89). Please see the corrected Table 9 here.

**Table 9:** Comparison of renal function amongst study participants (MDRD and CKD-EPI).

Parameter	Total (n = 162)	HIV-Controls (n = 52)	HIV-HAART (n = 111)	*p* value
***MDRD***				
Normal (eGFR≥90)	53 (32.70%)	22 (42.30%)	32 (28.8%)	0.1264
Mildly Reduced (eGFR 60-89)	93 (57.40%)	25 (48.10%)	68 (61.80%)	0.1256
Stage 3 (eGFR 30-59)	15 (9.30%)	5 (9.60%)	10 (9.10%)	1.0000
Stage 4 (eGFR 15-29)	1 (0.60%)	0 (0.00%)	1 (0.90%)	1.0000
Stage 5 (eGFR <15)	0 (0.00%)	0 (0.00%)	0 (0.00%)	−
***CKD-EPI***				
Normal (eGFR≥90)	99 (61.10%)	33 (63.50%)	67 (60.00%)	0.8365
Mildly Reduced (eGFR 60-89)	57 (35.20%)	17 (32.69%)	38 (34.50%)	0.8605
Stage 3 (eGFR 30-59)	5 (3.10%)	2 (3.85%)	5 (4.50%)	1.0000
Stage 4 (eGFR 15-29)	1 (0.60%)	0 (0.00%)	1 (0.90%)	1.0000
Stage 5 (eGFR <15)	0 (0%)	0 (0%)	0 (0%)	−

MDRD, Modification of Diet in Kidney Disease, CKD-EPI, Chronic Kidney Disease Epidemiology Collaboration. eGFR: estimated Glomerular Filtration Rate. n = Number. p values <0.05 were considered significant.
